# Thalidomide is more efficient than sodium butyrate in enhancing GATA-1 and EKLF gene expression in erythroid progenitors derived from HSCs with β-globin gene mutation

**Published:** 2016-01-01

**Authors:** Mohammad Ali Jalali Far, Ali Dehghani Fard, Saiedeh Hajizamani, Majid Mossahebi-Mohammadi, Hamid Yaghooti, Najmaldin Saki

**Affiliations:** 1 Health Research Institute, Research Center of Thalassemia and Hemoglobinopathy, Ahvaz Jundishapur University of Medical Sciences, Ahvaz, Iran; 2 Sarem Cell Research Center-SCRC, Sarem Women’s Hospital, Tehran, Iran; 3 Diagnostic Laboratory Sciences and Technology Research Center, School of Paramedical Sciences, Shiraz University of Medical Sciences, Shiraz, Iran; 4 Department of Hematology, Faculty of Medical Sciences, Tarbiat Modares University, Tehran, Iran; 5 Department of Medical Laboratory Sciences, School of Paramedicine, Ahvaz Jundishapur University of Medical Sciences, Ahvaz, Iran

**Keywords:** Fetal hemoglobin, Thalidomide, Sodium butyrate, β-thalassemia

## Abstract

**Background:** Efficient induction of fetal hemoglobin (HbF) is considered as an effective therapeutic approach in beta thalassemia. HbF inducer agents can induce the expression of γ-globin gene and produce high levels of HbF via different epigenetic and molecular mechanisms. Thalidomide and sodium butyrate are known as HbF inducer drugs.

**Material and methods**: CD133^+^ stem cells were isolated from umbilical cord blood of a newborn with minor β-thalassemia in order to evaluate the effects of these two drugs on the in vitro expression of GATA-1 and EKLF genes as erythroid transcription factors. CD133^+^ stem cells were expanded and differentiated into erythroid lineage and then treated with thalidomide and sodium butyrate and finally analyzed by quantitative real-time PCR. Statistical analysis was performed using student’s t-test by SPSS software.

**Results**: Thalidomide and sodium butyrate increased GATA-1 and EKLF gene expression, compared to the non-treated control (P<0.05).

**Conclusion**: Thalidomide was more efficient than sodium butyrate in augmenting expression of GATA-1 and EKLF genes. It seems that GATA-1 and EKLF have crucial roles in the efficient induction of HbF by thalidomide.

## Introduction

 Beta Thalassemia (β-thalassemia) is one of the most common genetic disorders of hemoglobine chain synthesis caused by reduced or absence of β-globin chain production. Following these defects, additional alpha chains (α – chains) precipitates in erythroid precursors and lead to inefficient erythropoiesis and membrane damage.^1^^-^^3^ Recently, induction of hemoglobin F (HbF) expression has been proposed as a novel therapeutic approach to improve clinical and pathological features of inefficient erythropoiesis in patients with β-thalassemia.^4^ It has been shown that induction of HbF expression leads to reduction of accumulation of additional α- globin chains in erythroid precursors, resulting in betterment of the imbalance between α and β chains. Consequently, this therapy diminishes ineffective erythropoiesis.^5^ HbF inducer agents consist of immunomodulators such as thalidomide^6^ and pomalidomide,^7^ histone deacetylase enzyme inhibitors (HDAC) such as 5–Aza^8^ and decitabine,^9^ butyrate derivatives,^10^ cytotoxic/(hypomethylating drugs such as hydroxyurea^11^ and recombinant human erythropoietin (rhEPO).^12^ GATA-1 and erythroid Krupple-like factor (EKLF) are two important and specific transcription factors in erythroid differentiation which play critical roles in regulation of globin gene expression. These transcription factors alter globin gene expression by affecting the promoter regions as well as locus control region (LCR).^13^ It has been shown that GATA-1 augments gene expression by means of increasing H3K4di- and trimethylation of β – globin gene.^14^

In the present study, thalidomide and sodium butyrate were used as gamma (γ) – globin gene inducers in order to evaluate their mechanism in inducing gene expression. Regarding the higher ability of thalidomide in comparison to sodium butyrate to induce γ-globin gene expression in vitro at various concentrations^15^^,^^16^ and also to induce higher levels of erythroid differentiation,^17^ this work aimed to evaluate and compare these drugs in changing the pattern of gene expression of erythroid transcription factors GATA-1 and EKLF. The study is designed to better understand the mechanism of these two drugs in inducing HbF expression. This is the first study that evaluates the molecular mechanisms of HbF induction using an umbilical cord blood sample from a minor β-thalassemia newborn.

## SUBJECTS AND METHODS

**Drugs and Erythroid Growth factors**

 In the present study, recombinant human erythropoietin (rhEPO, R&D systems, Minneapolis, MN, USA), interleukin–3 (IL-3, Stem Cell Technologies and Vancouver, BC, Canada), thalidomide (Tocris Bioscience, Missouri, USA) and sodium butyrate (Sigma, Saint Louis, MO, USA) were used in order to induce gene expression and erythroid differentiation.

**Mononuclear cell isolation from umbilical cord blood**

Umbilical cord blood (UCB) samples were collected from a newborn with minor β-thalassemia following full-term delivery. Informed consent was obtained from the parents (Sarem Hospital, Tehran, and ethical No: ajums.REC.1392.160). Blood samples were collected into blood bags containing sodium citrate diluted with hydroxyethyl starch (HES) in the ratio of 1:6 to deplete red blood cells (RBCs).The diluted samples were layered onto a Ficoll- Paque (Amersham Pharmacia, Piscataway, NJ, ρ= 1.077 g/mL) gradient and centrifuged at 400 × g for 20 minutes at 24⁰C. Following centrifugation, mononuclear (MNC) interface layer was recovered and washed twice with phosphate-buffered saline (PBS) / EDTA.

**Isolation of CD133** ^+^ **cells**

CD133^+^ cells were isolated from MNSCs by magnetic activated cell sorting (MACS) (Miltenyi Biotech, Germany) according to manufacturer's instructions. MNCs were incubated with 50 µl of CD133 microbeads (conjugated to iron particles) (IQ-Products, Groningen, The Netherlands) for 30 minutes at 4⁰C in dark. Cells were then washed with PBS containing 0.5% bovine serum albumin (BSA; Sigma-Aldrich, St. Louis, MO, USA) and 2 mM EDTA. Positive selection was done by passage through LS columns. Purified CD133^+^ cells were cultivated in StemSpan Culture Medium (Stem Cell Technologies, Vancouver, BC, Canada) containing defined growth factors. Purity of isolated CD133^+^ cells was assessed by flow cytometric analysis. Purity of the isolated cells was above 95 %.

**Cell culture, Erythroid Differentiation and Drug treatment**

Isolated CD133^+^ cells were cultured in Iscove's modified Dulbecco's medium (IMDM; Sigma-Aldrich , St. Louis, MO, USA) supplemented with fetal bovine serum (FBS; Cambrex, Belgium), 2 mM L- glutamine (Sigma – Aldrich, St. Louis, MO, USA) and 100 U/ml penicillin / streptomycin (Sigma – Aldrich, St. Louis, MO, USA). Expanded CD133^+^ cells were differentiated into erythroid lineage using 4 U/ml rhEPO and 5 ng/ml IL-3. On the 6^th^ day of differentiation, cells were divided into four different groups. The studied groups were as follows: (1) non-treated cells at day 6 as baseline control group; (2) group treated with 100 µM thalidomide; (3) sodium butyrate 100 µM; and (4) 0.1% dimethylsolfoxide (DMSO; Sigma – Aldrich, St. Louis, MO, USA) as vehicle control group. Cells were provided with fresh medium every 72 hours. Finally, treated erythroid cells were harvested at days 9 or 12 of incubation.

**RNA isolation and Real time PCR**

To analyze GATA-1 and EKLF gene expression, qRT-PCR technique was performed at days 6, 9 and 12 of differentiation. Total RNA was extracted from cells using RNeasy mini kit (Qiagen, Valencia, CA). Reverse transcription (RT) was performed using RevertAid kit (Fermentas, Lithuania). Relative quantiﬁcation of GATA-1 and EKLF expression was performed by the StepOne Plus Real-Time RT-PCR System (Applied Biosystems, USA) using SYBR Green Master Mix (Fermentas, Lithuania) according to the manufacturer's instructions. Primer sequences are shown in Table 1. The data were analyzed by 2 ^–∆∆CT^ method and are presented as fold change in gene expression. Normalization was performed against β-actin as housekeeping gene. All samples were run in triplicates.

**Statistical analysis**

Results are shown as mean ± SD of independent measurements. Statistical analysis was performed using student’s t-test by SPSS software (v.13.0). Probability of P< 0.05 was considered statistically significant.

**Table 1:** The sequences of the primers used for qRT-PCR

**Primers**	**Forward Sequence**	**Reverse Sequence**
** GATA-1**	5´-AGACGACCACCACGACAC- 3´	5´-CCAGATGCCTTGCGGTTTC- 3´
**EKLF**	5´-CGCCTTGCCCTCCATCAG- 3´	5´-CCCTCTCATCGTCCTCTTCC- 3´
**β-actin **	5´-CCCTGGCGGCCAAGGACTC-3´	5´-CACATGCCGGAGCCGTTGTC- 3´

## Results

**Figure-1:**
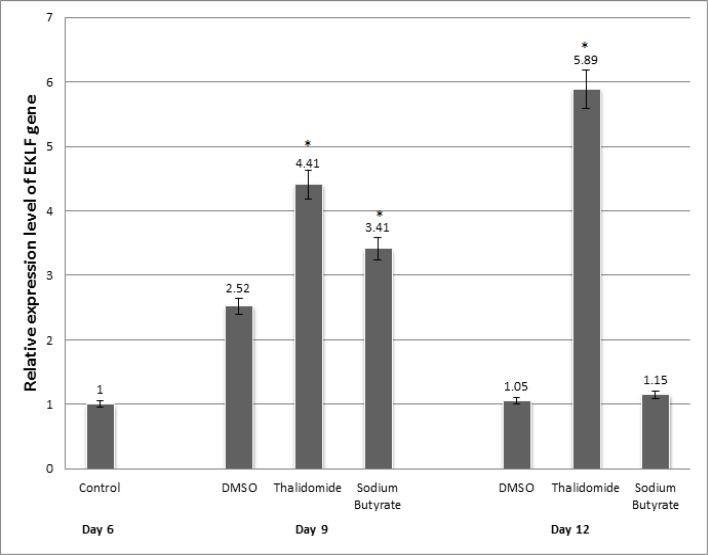
EKLF gene expression during erythroid differentiation in the presence of drugs. On the 6th day of differentiation, cells were divided into four different groups. The studied groups were as follows: non-treated cells at day 6 as baseline control group; group treated with 100 μM thalidomide; sodium butyrate 100 μM; and 0.1% dimethylsolfoxide (DMSO) at days 9 and 12 of erythroid differentiation. Results are expressed with relative to non-treated control group/ as Mean ± SD (n = 3).* p<0.05 vs. control group.

**Figure-2:**
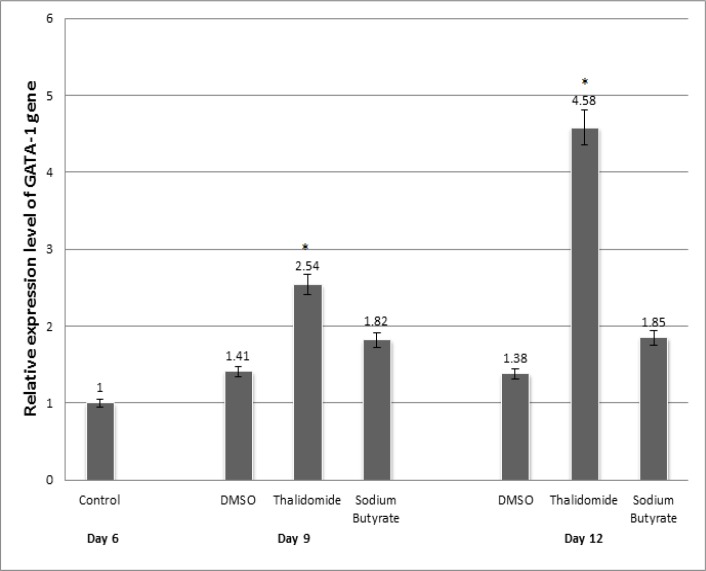
GATA-1 gene expression during erythroid differentiation in the presence of drugs.

 To evaluate and compare the effects of thalidomide and sodium butyrate on altering the expression pattern of GATA-1 and EKLF, isolated CD133^+ ^cells were treated with thalidomide and sodium butyrate and finally analyzed by quantitative real-time PCR. As shown in figure 1, EKLF gene expression showed 4.41- and 5.89-fold increase in thalidomide group compared to controls at days 9 and 12 of erythroid differentiation, respectively (P< 0.05). The increases in EKLF gene expression were 3.41 and 1.15 fold in sodium butyrate group compared to controls at days 9 and 12 of erythroid differentiation, respectively (P< 0.05). As also shown in figure 2, our results showed 2.54- and 4.58-fold increase in GATA-1 gene expression in thalidomide group compared to control at days 9 and 12 of erythroid differentiation, respectively (P< 0.05). The increases in GATA-1 gene expression were 1.82-and 1.85-fold in sodium butyrate group compared to control at days 9 and 12 of erythroid differentiation, respectively (P< 0.05). Our results confirmed that thalidomide was more efficient than sodium butyrate in EKLF and GATA-1 induction.

## Discussion

 Thalidomide and sodium butyrate are effective drugs in inducing HbF expression with fewer side effects compared to other drugs.^6^^,^^10^ As previously reported, thalidomide is a potent agent in enhancing proliferation of erythroid precursors,^17^ HbF up-regulation,^15^ and decreasing H3K27 methylation of γ-globin gene promoter.^18^

Therefore, investigation of alterations in expression of erythroid transcription factors would help clarify the molecular mechanisms involved in induction of HbF. According to prominent role of transcription factors GATA-1 and EKLF in erythroid differentiation and induction of globin gene expression, the association between changes in their expression and induction of HbF expression was studied. Our results indicated that the up-regulation in EKLF and GATA-1 expression in response to thalidomide and sodium butyrate is in accordance with induction of HbF expression. These findings suggest that GATA-1 and EKLF are implicated in inducing HbF expression. We confirmed that thalidomide was more efficient than sodium butyrate in inducing GATA-1 and EKLF (P< 0.05). According to previous studies, thalidomide is more efficient than sodium butyrate in inducing γ-globin gene expression.^15^^,^^16^ It seems that there is a close association between increasing in expression of GATA-1 and EKLF with γ-globin gene expression.

## CONCLUSION

 Considering the fact that effective production of HbF results in reduction of clinical complications of β-thalassemia, it appears that effective up-regulation of GATA-1 and EKLF expression using these medications could lead to effective induction of HbF expression and healing of patients. Examining other molecular and epigenetic mechanisms involved in expression of globin genes could help identify other means of augmenting HbF production.^19^

On the 6th day of differentiation, cells were divided into four different groups. The studied groups were as follows: non-treated cells at day 6 as baseline control group; group treated with 100 μM thalidomide; sodium butyrate 100 μM; and 0.1% dimethylsolfoxide (DMSO) at days 9 and 12 of erythroid differentiation. Results are expressed with relative to non-treated control group/ as Mean ± SD (n = 3).* p<0.05 vs. control group.
